# Supplementary material for: Clinical Grade Human Pluripotent Stem Cell-Derived Engineered Skin Substitutes Promote Keratinocytes Wound Closure In Vitro
Source: Cells. 2022 Mar 29;11(7):1151. doi: 10.3390/cells11071151 (PMC8998132; doi:10.3390/cells11071151)
Supplement: Supplementary file 1 [file cells-11-01151-s001.zip › cells-1603311 supplementary.pdf]

cells

MDPI

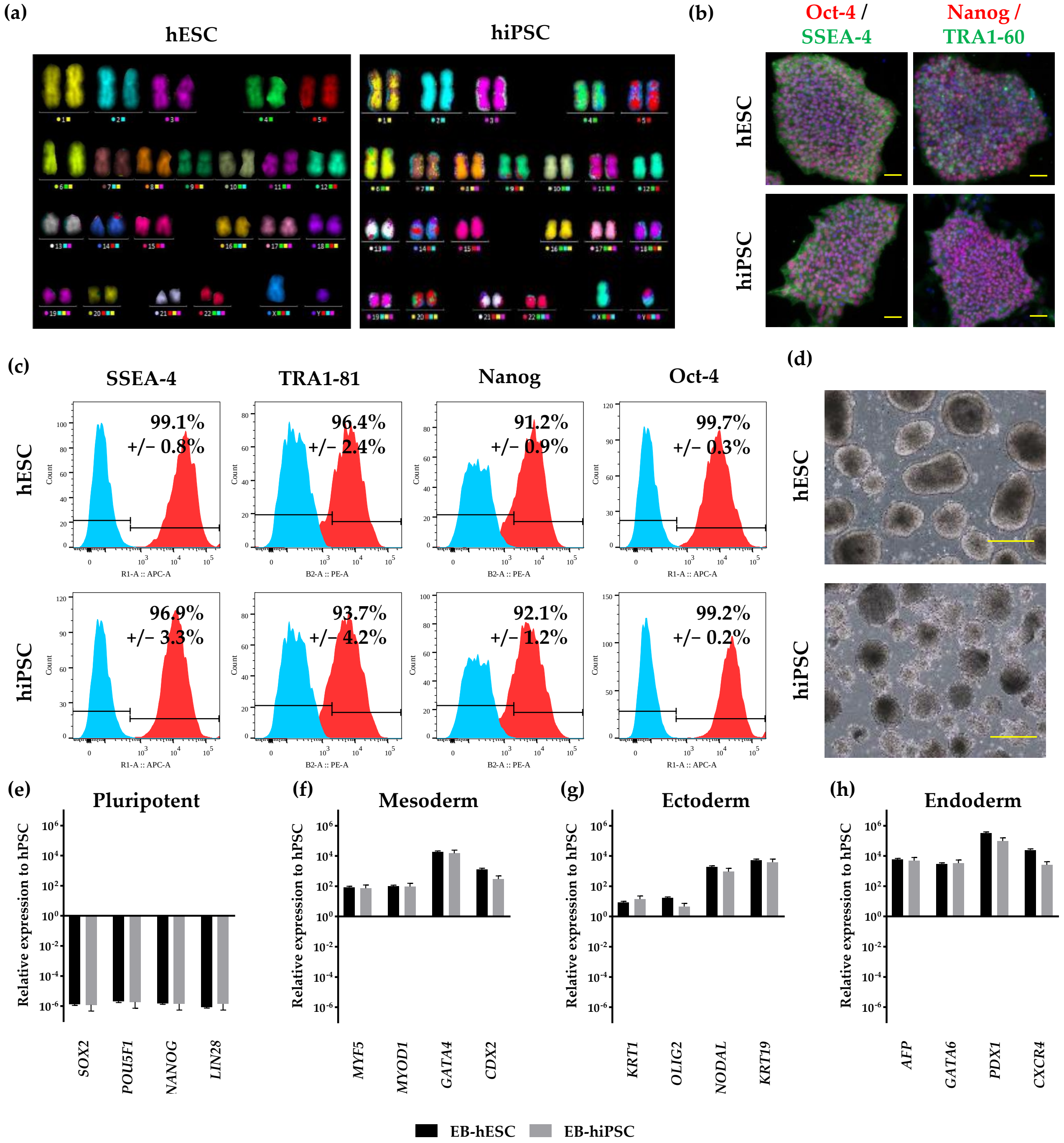

Figure S1. Characterization of hESC RC9 and hiPSC PC1432. (a) Karyotype analysis by mFISH staining of hESC RC-9 (46:XY) and hiPSC PC-1432 (46:XY). (b) Microscopic analysis of pluripotency membrane markers SSEA-4, TRA1-60, and nuclear markers Nanog, Oct-4 (Scale bars : 50µm). (c) Flow cytometry analysis of SSEA-4, TRA1-81, Nanog and Oct-4 expression (in red). Staining with isotypic antibody was performed as control (in blue). (d) Microscopic analysis of embryoïd bodies (EB) derived from hPSCs after 14 days of culture (Scale bars : 200µm). (e-h) Pluripotency and three germ layers markers analysis by RTq-PCR in EB derived from hPSCs after 14 days of differentiation. Results are expressed as mean ± SD (n ≥ 3 independent experiments).

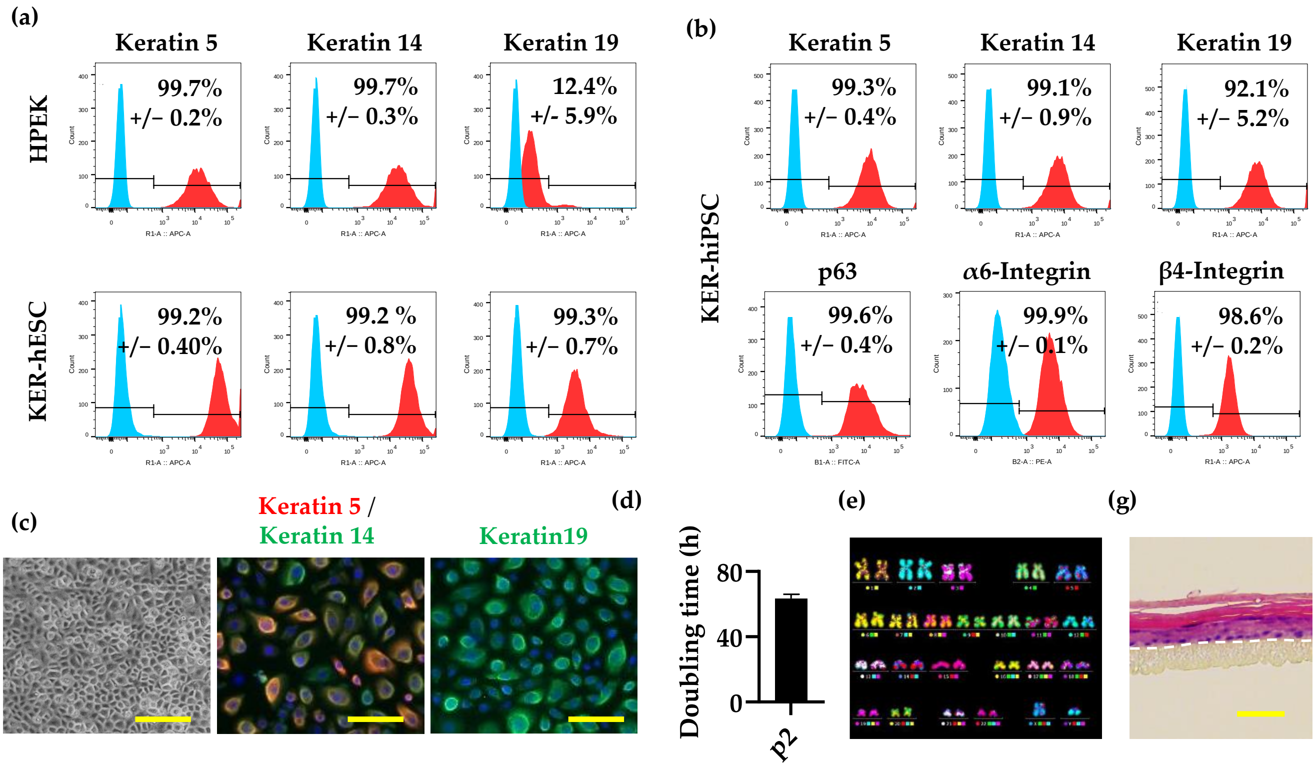

Figure S2. Characterization of a homogeneous and functional population of keratinocytes derived from human pluripotent stem cells. (a-b) Flow cytometry analysis of keratin 5, keratin 14 and keratin 19 in HPEK, KER-hESC and KER-hiPSC p63, α6-Integrin and β4-Integrin expression KER-hiPSC (in red). Staining with isotypic antibody (in blue) was performed as control. (c) Microscopic observation of KER-hiPSCp2 morphology (Scale bars : 200µm) and immunocytochemistry analysis of keratin 5, keratin 14 and keratin 19 in KER-hiPSC (Scale bars : 100µm). (e) Doubling time of KER-hiPSCp2. (f) Karyotype analysis by mFISH staining of KER-iPSC (46:XY). (g) Eosin-Hematoxylin staining of epidermal reconstitution on polycarbonate membrane (Scale bars: 100 µm).

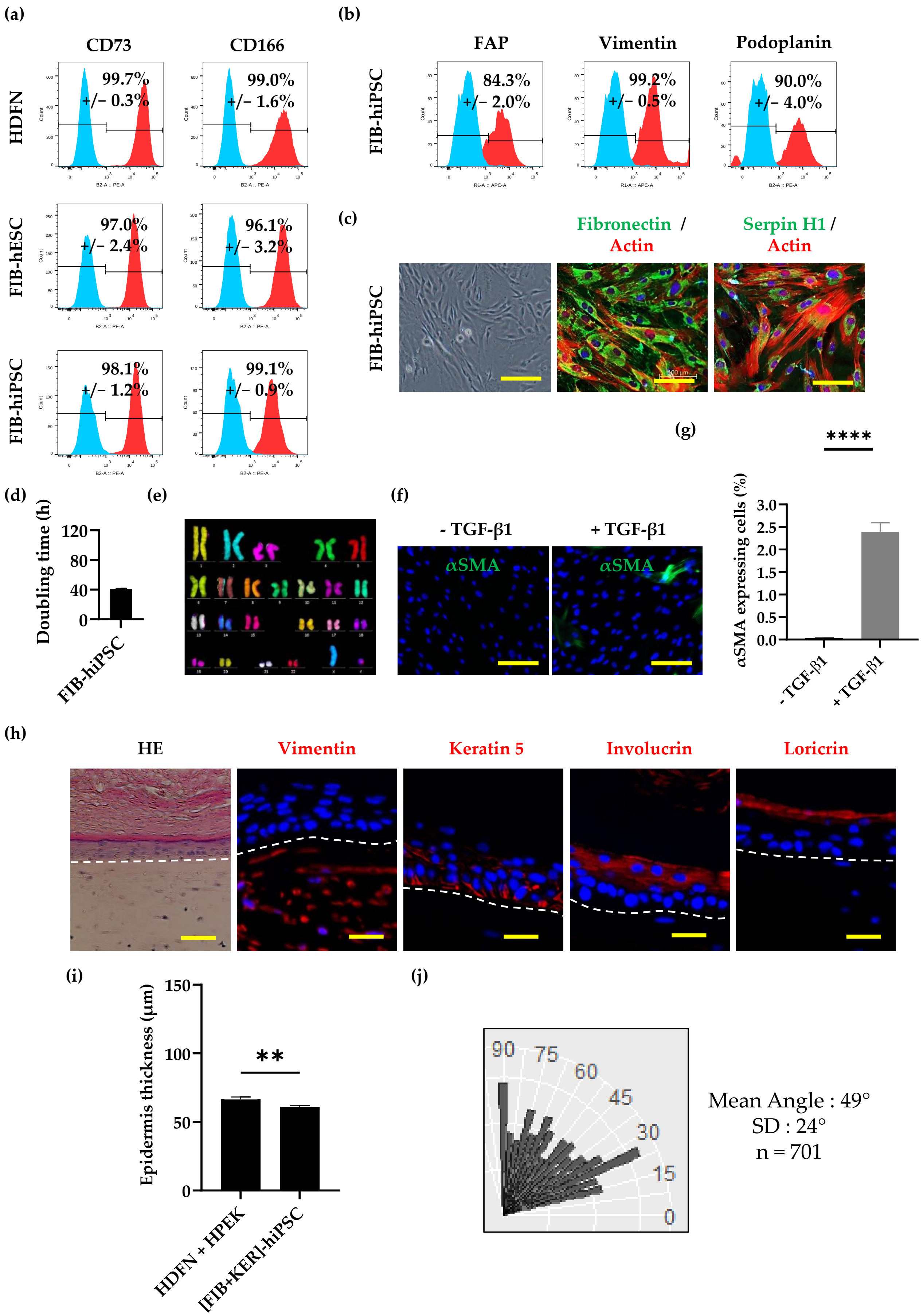
(a)
CD73
CD166
HDFN
99.7% +/− 0.3%
99.0% +/− 1.6%
FIB-hESC
97.0% +/− 2.4%
96.1% +/− 3.2%
FIB-hiPSC
98.1% +/− 1.2%
99.1% +/− 0.9%
(b)
FAP
Vimentin
Podoplanin
FIB-hiPSC
84.3% +/− 2.0%
99.2% +/− 0.5%
90.0% +/− 4.0%
(c)
Fibronectin / Actin
Serpin H1 / Actin
FIB-hiPSC
(d)
Doubling time (h)
120
80
40
0
FIB-hiPSC
(e)
(f)
- TGF-β1
+ TGF-β1
αSMA
αSMA
(g)
****
αSMA expressing cells (%)
3.0
2.5
2.0
1.5
1.0
0.5
0.0
- TGF-β1
+ TGF-β1
(h)
HE
Vimentin
Keratin 5
Involucrin
Loricrin
(i)
Epidermis thickness (µm)
150
100
50
0
**
HDFN + HPEK
[FIB+KER]-hiPSC
(j)
90
75
60
45
30
15
0
Mean Angle : 49°
SD : 24°
n = 701

Figure S3: Characterization of a homogeneous and functional population of fibroblasts derived from human pluripotent stem cells. (a-b) Flow cytometry analysis of CD73 and CD166 markers in HDFN, FIB-hESC and FIB-hiPSC and FIBRO, FAP, Vimentin and Podoplanin in FIB-hiPSC (in red). Staining with isotypic antibody (in blue) was performed as control. (c) Microscopic observation of FIB-hiPSC morphology (Scale bars : 200µm) and immunocytochemistry analysis of Serpin H1 and Fibronectin (Scale bars : 100µm). Cell's shape is observed with actin staining. (d) Doubling time of at p3. (e) Karyotype analysis by mFISH staining of FIB-iPSC (46:XY). (f-g) Immunocytochemistry analysis and associated quantification of αSMA in differentiated HDFN and FIB-hESC after TGF-β1 stimulation. (Scale bars: 200 µm). For statistical significance: **** $p < 0.001$. (h) Epidermal reconstitution of keratinocytes on plasma based matrix containing fibroblasts analysis by Haematoxylin-Eosin staining (Scale bars : 100µm) and immunohistochemistry analysis (Scale bars : 50µm) of dermal marker vimentin, epidermal basal layer marker keratin 5 and suprabasal markers involucrin and loricrin. (i) Epidermis thickness measurement between the basal and beginning of the corneal layer (µm). (j) Distribution of basal keratinocyte nuclei according to angle versus the JDE plan into angle categories from 0° to 90°, characterized by automated image analysis. The vertical axis represents angle values and the horizontal axis numbers of cells in the different angle categories. n correspond to the number of analyzed nuclei.

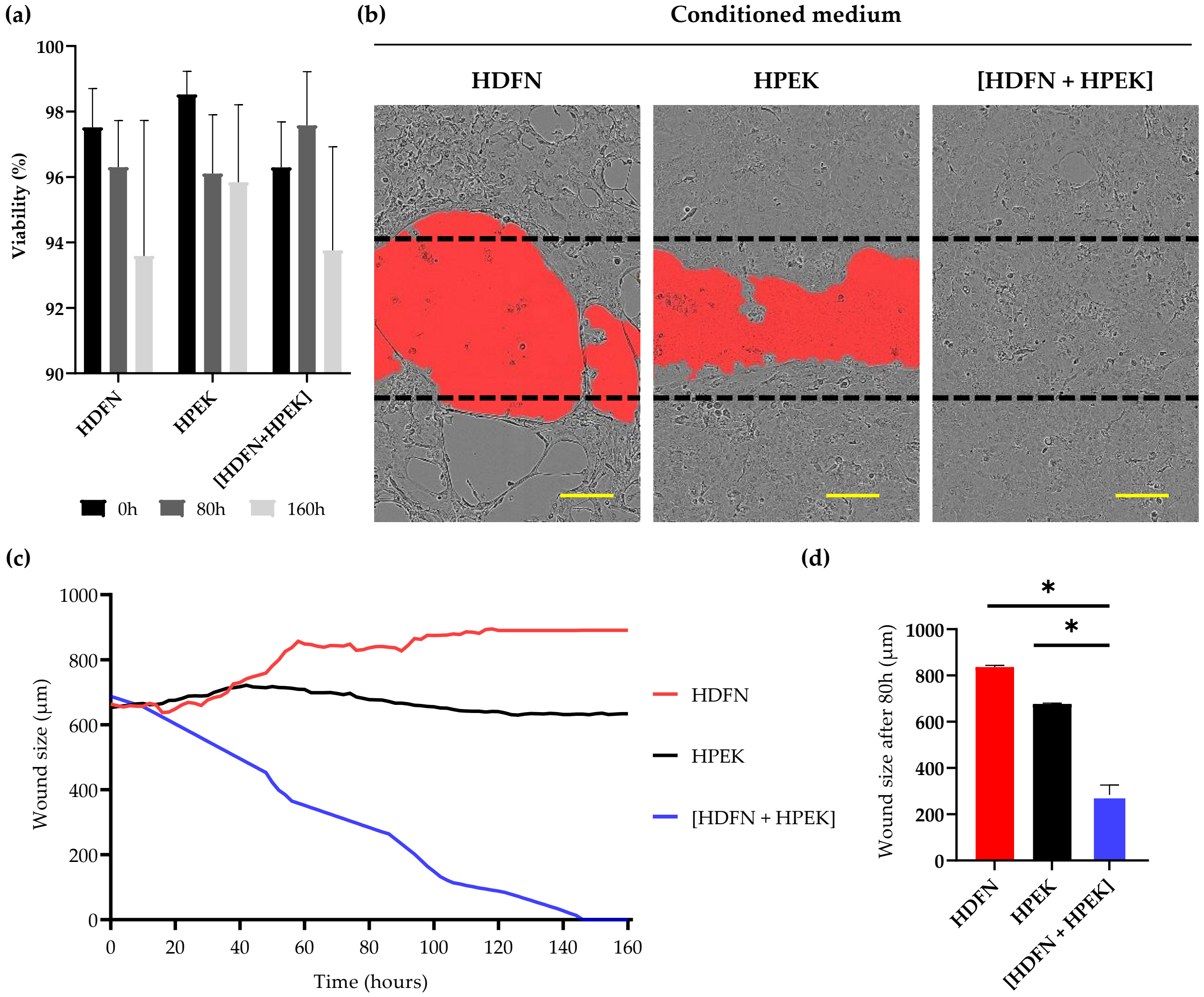

Figure S4. Conditioned medium from primary cells dermo-epidermal reconstituted tissue allows WT wound closure *in vitro*. (a) Viability measured on keratinocyte monolayer with Incucyte® device and software. (b) Monitoring of in vitro wound closures during 160h with conditioned media. (c) Wounds appearance 160h after conditioned media treatments (Scale bars : 200µm). (d) Comparison of keratinocyte wounds size of scratch areas 80h after the beginning of the experiment. For statistical significance, * $p < 0.05$.

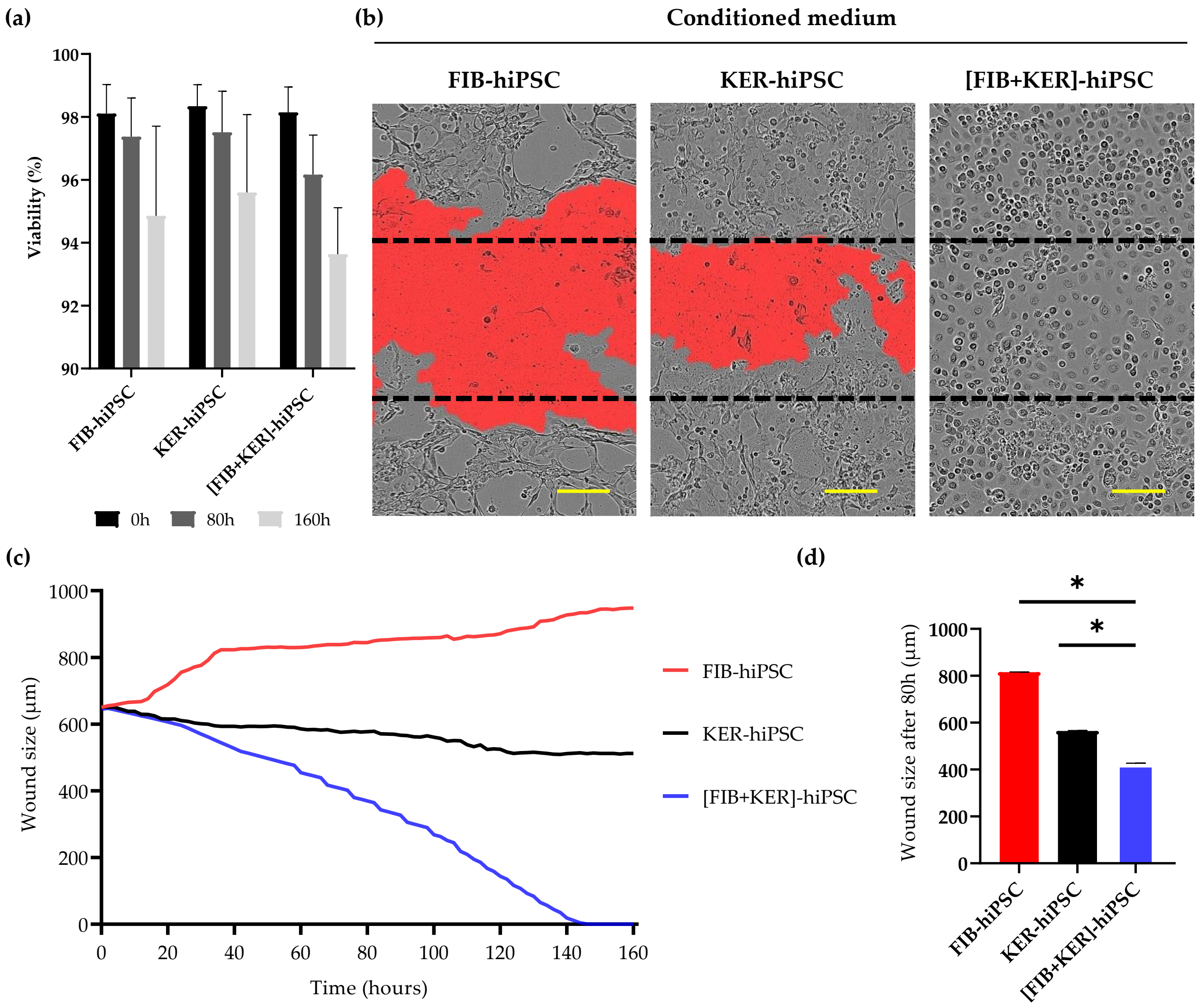

Figure S5. Conditioned medium from hiPSC derived dermo-epidermal reconstituted tissue allows WT wound closure in vitro. (a) Viability measured on keratinocyte monolayer with Incucyte® device and software. (b) Monitoring of in vitro wound closures during 160h with conditioned media. (c) Wounds appearance 160h after conditioned media treatments (Scale bars : 200µm). (d) Comparison of keratinocyte wounds size of scratch areas 80h after the beginning of the experiment. For statistical significance, * $p < 0.05$.

Table S1 : Primary cells used in this study

Cell name	Provider	Reference	Gender of donor	Age	Ethnicity	Original tissue
HPEK	CELLnTEC	HPEKs	Male	Juvenile	Caucasian	Foreskin
HDFN	Cell Applications	106-05n	Male	Neonatal	Caucasian	Foreskin

Table S2. Antibodies references used in this study

Markers	References	Providers	Use
OCT-3/4	2890	Cell Signaling	ICC
	130-105-555	Miltenyi Biotec	FC
SSEA-4	4755	Cell Signaling	ICC
	FAB1435A	R&D System	FC
Nanog	4903	Cell Signaling	ICC
	130-105-050	Miltenyi Biotec	FC
TRA1-60	4746	Cell Signaling	ICC
TRA1-81	FAB8495P	R&D System	FC
Keratin 5	ab52635	Abcam	ICC / IHC
Keratin 14	905301	Biolegend	ICC
Keratin 19	sc-376126	SANTA CRUZ	ICC
p63	ab246727	Abcam	FC
α6-Integrin	130-119-767	Miltenyi Biotec	FC
β4-Integrin	130-124-246	Miltenyi Biotec	FC
Serpin H1	NBP1-97491	NOVUS Biologicals	ICC
Fibronectin	sc-18825	SANTA CRUZ	ICC
FAP	FAB3715A	R&D System	FC
CD73	550257	BD-Biosciences	FC
CD166	559263	BD-Biosciences	FC
α-SMA	14-9760-82	INVITROGEN	ICC
Vimentin	sc-373717	SANTA CRUZ	IHC
	ab176512	Abcam	FC
Popoplanin	127408	Biolegend	FC
Involucrin	I9018-.2ML	Sigma Aldrich	IHC
Loricrin	905101	Biolegend	IHC
Goat anti-Rabbit IgG, Alexa Fluor 555	A21428	INVITROGEN	ICC / IHC
Goat anti-Mouse IgG, Alexa Fluor 555	A21424	INVITROGEN	ICC / IHC
Goat anti-Rabbit IgG, Alexa Fluor 488	A11008	INVITROGEN	ICC / IHC
Goat anti-Mouse IgG, Alexa Fluor 488	A32723	INVITROGEN	ICC / IHC

Table S3. Genes analyzed with Taqman array plate

Embryonic sheet	Gene Symbol	Assay ID
Pluripotent markers	GABRB3	Hs00241459_m1
	GDF3	Hs00220998_m1
	TDGF1	Hs02339499_g1
	UTF1	Hs00864535_s1
	ZFP42	Hs01938187_s1
	SOX2	Hs01053049_s1
	POU5F1	Hs00999632_g1
	NANOG	Hs02387400_g1
	LIN28	Hs00702808_s1
	DNMT3B	Hs00171876_m1
Mesoderm markers	MYF5	Hs00929416_g1
	MYOD1	Hs00159528_m1
	GATA4	Hs00171403_m1
	CDX2	Hs01078080_m1
Ectoderm markers	KRT1	Hs00196158_m1
	OLIG2	Hs00377820_m1
	NODAL	Hs00415443_m1
	KRT19	Hs00761767_s1
Endoderm markers	AFP	Hs00173490_m1
	GATA6	Hs00232018_m1
	PDX1	Hs00426216_m1
	CXCR4	Hs00607978_s1
Housekeeping genes	18s rRNA	Hs99999901_s1
	GAPDH	Hs99999905_m1
	HPRT	Hs99999909_m1
	GUSB	Hs99999908_m1

Table S4. Culture conditions from previously described differentiation's protocols

Bibliography	hES culture conditions		Diffentiation conditions
This study	Feeder : no<br>Coating : L7 matrix<br>Medium : StemPro hES SFM, FGF2	FIB	Method/coating : hPSC clumps on L7 matrix<br>Media : CnT-PR-F, defined FBS<br>Cytokines : BMP-4
		KER	Method/coating : hPSC colony clumps on L7 matrix, collagen I<br>Media : D-KSFM, CnT-07-HC<br>Cytokines : RA, BMP-4
Shamis et al. (2011)	Feeder : MEF feeder layer*<br>Coating : gelatin<br>Medium : KO-DMEM, KSR, GlutaMax-I, MEM NEAA, FGF2	FIB	Method : hESC clumps on MEF feeder layer*, gelatin, collagen I<br>Media : DMEM/F12, FBS, adenine*, HC, cholera toxin*, EGF, insulin<br>Cytokines : BMP-4
Itoh et al. (2013)	Feeder : MEF feeder layer*<br>Coating : gelatin<br>Medium : KO-DMEM, KSR, GlutaMax-I, MEM NEAA, FGF2	FIB	Method/coating : EB from hiPSC on gelatin<br>Media : DMEM + FBS<br>Cytokines : Ascorbic acid (AA), TGF-β2, ITS-A*
		KER	Method/coating : hiPSC clumps on matrigel*, fibronectin, collagen I<br>Media : D-KSFM, CnT-07<br>Cytokines : RA, BMP-4
Kim et al. (2018)	Feeder : no<br>Coating : vitronectin<br>Medium : TeSR-E8	FIB	Method/coating : EB from hPSC on matrigel*, collagen I<br>Media : DMEM-F12, FBS, adenine*, insulin, EGF, MEM NEAA<br>Cytokine : BMP-4
		KER	Method/coating : EB from hPSC on collagen IV*<br>Media : TeSR-E8, DMEM/F12, FBS, AA, adenine*, insulin, KSFM*, D-KSFM<br>Cytokines : RA, BMP-4, EGF
Soares et al. (2019)	Feeder : no<br>Coating : geltrex*<br>Medium : E8 Flex	KER	Method/coating : single cell hPSC on geltrex*<br>Media : KSFM*, EGF, BPE*,<br>Cytokines : RA, BMP-4
Jacków et al. (2019) **	Feeder : no<br>Coating : vitronectin<br>Medium : E8, revitacell	FIB	Method/coating : EB from hiPSC on gelatin<br>Media : DMEM + FBS<br>Cytokines : AA
		KER	Method/coating : hiPSC clumps on vitronectin<br>Media : D-KSFM<br>Cytokines : RA, BMP-4
Zhang et al. (2019)	Feeder : no<br>Coating : matrigel*<br>Medium : E8	FIB	Method/coating : hiPSC clumps on matrigel*<br>Media : RPMI*, B27, IWR-1*, advanced DMEM<br>Cytokines : CHIR99021, RA, FGF2, SB431542
		KER	Method/coating : hPSC clumps on matrigel*<br>Media : DMEM-F12, AA, ITS*, chemically defined lipid concentrate*, adenine*, BSA, HC, NKH477, $MgCl_2$, $MgSO_4$, L-$NaHCO_3$, L-glutamine, L-Leucine, L-lysine monohydrochloride, L-methionine and CaCl2<br>Cytokines : CHIR99021, DAPT, EGF, SB431542, BMP-4
Sah et al. (2021)	Feeder : no<br>Coating : matrigel*<br>Medium : PeproGrow hES medium*	FIB	Method/coating : EB from hiPSC on gelatin<br>Media : DMEM + FBS<br>Cytokines : AA, TGFβ2, ITS-A*
		KER	Method : single cell hPSC on matrigel*<br>Media : D-KSFM, CnT-07<br>Cytokines : RA, BMP-4

*Not GMPc product, ** Jacków, J.; Guo, Z.; Hansen, C.; Abaci, H.E.; Doucet, Y.S.; Shin, J.U.; Hayashi, R.; DeLorenzo, D.; Kabata, Y.; Shinkuma, S.; et al. CRISPR/Cas9-Based Targeted Genome Editing for Correction of Recessive Dystrophic Epidermolysis Bullosa Using IPS Cells. *Proc. Natl. Acad. Sci. USA* **2019**, 116, 26846–26852. https://doi.org/10.1073/pnas.1907081116.
